# Impact of Interleukin-18 Polymorphisms -607A/C and -137G/C on Oral Cancer Occurrence and Clinical Progression

**DOI:** 10.1371/journal.pone.0083572

**Published:** 2013-12-13

**Authors:** Hsiu-Ting Tsai, Chung-Han Hsin, Yi-Hsien Hsieh, Chih-Hsin Tang, Shun-Fa Yang, Chiao-Wen Lin, Mu-Kuan Chen

**Affiliations:** 1 School of Nursing, Chung Shan Medical University, Taichung, Taiwan; 2 Department of Nursing, Chung Shan Medical University Hospital, Taichung, Taiwan; 3 School of Medicine, Chung Shan Medical University, Taichung, Taiwan; 4 Department of Otolaryngology, Chung Shan Medical University Hospital, Taichung, Taiwan; 5 Department of Biochemistry, School of Medicine, Chung Shan Medical University, Taichung, Taiwan; 6 Graduate Institute of Basic Medical Science, China Medical University, Taichung, Taiwan; 7 Department of Biotechnology, College of Health Science, Asia University, Taichung, Taiwan; 8 Institute of Medicine, Chung Shan Medical University, Taichung, Taiwan; 9 Institute of Oral Sciences, Chung Shan Medical University, Taichung, Taiwan; 10 Department of Dentistry, Chung Shan Medical University Hospital, Taichung, Taiwan; 11 Department of Otorhinolaryngology-Head and Neck Surgery, Changhua Christian Hospital, Changhua, Taiwan; Sudbury Regional Hospital, Canada

## Abstract

**Background:**

The purpose of this study was to identify gene polymorphisms of interleukin-18 (IL-18) -607A/C and -137G/C specific to patients with oral cancer susceptibility and clinicopathological status.

**Methodology and Principal Findings:**

A total of 1,126 participants, including 559 healthy people and 567 patients with oral cancer, were recruited for this study. Allelic discrimination of *-607A/C* (rs1946518) and *-137G/C* (rs187238) polymorphisms of the *IL-18* *gene* was assessed by a real-time PCR with the TaqMan assay. There was no significant association between *IL-18 -607A/C* polymorphism and oral cancer risk. However, among alcohol consumers, people with *A/A* *homozygotes* of *IL-18 -607A/C* polymorphism had a 2.38-fold (95% CI=1.17-4.86; p=0.01) increased risk of developing oral cancer compared with those with *C/C* *homozygotes*. The participants with *G/C* *heterozygotes* of *IL-18 -137* *polymorphism* had a 1.64-fold (95% CI: 1.08-2.48; p=0.02) increased risk of developing oral cancer compared with those with *G/G* wild type homozygotes. Both sets of statistics were determined after adjusting for confounding factors. Among people who had exposure to oral cancer-related environmental risk factors such as areca, alcohol, and tobacco consumption, the adjusted odd ratios and 95% confidence intervals were increased to a 2.02-fold (95% CI=1.01-4.04; p=0.04), 4.04 (95% CI=1.65-9.87; p=0.002) and a 1.66-fold (95% CI=1.00-2.84; p=0.05) risk of developing oral cancer. However, patients with *G/C* alleles of *IL-18 -137* were correlated with a lower clinical stage (AOR=0.59; 95% CI=0.39-0.89; p=0.01), smaller tumor size (AOR=0.56; 95% CI=0.35-0.87; p=0.01), and non-lymph node metastasis (AOR=0.51; 95% CI=0.32-0.80; p=0.003).

**Conclusion:**

*IL-18 -137* *G/C* gene polymorphism may be a factor that increases the susceptibility to oral cancer, as well as a protective factor for oral cancer progression. The interactions of gene to oral cancer-related environmental risk factors have a synergetic effect that can further enhance oral cancer development.

## Introduction

Oral cancer is malignant and usually causes extensive damage to the organs involved [1]. In Taiwan, the incidence rate of oral cancer is 20.22/100,000, and it is the 7^th^ prevalent malignancy [2] and the 5^th^ leading cause of cancer deaths among Taiwanese [3]. *Interleukin-18* (*IL-18*), an 18-kDa cytokine, belongs to the interleukin-1 (IL-1) superfamily and is produced by various immune and non-immune cells [4-7]. It has been demonstrated that the expression and secretion of *IL-18* is a crucial event against oncogenesis of oral carcinoma cells because of its modulation of cell cycle progression or its triggering of an apoptotic pathway [4,6-8]. Nilkaeo et al. found that the *IL-18* suppressed KB cell line, a carcinoma cell line derived from oral cavity, proliferates in a dose-dependent manner through the modulation of cell-cycle arrest in the S phase [6]. Liu et al. demonstrated that the over-expression of *IL-18* reduced cell viability and induced apoptosis of the human tongue squamous cell carcinoma cell line could be attributed to the down-regulation of cyclin D1 expression and a caspase-dependent pathway, respectively [7]. We suggested that *IL-18* is a key regulator for the development of oral cancer. 

The *IL-18* gene is located on chromosome 11q22. Two functional gene polymorphisms *-607A/C* and *-137G/C* are found in its promoter region [9]. Giedraitis et al. analyzed *IL-18* gene promoter sequence and found a change from *C* allele to *A* allele at position -607 and a change from *G* to *C* at position -137 of the *IL-18* promoter region [9]. They estimated transcription activity of *IL-18* gene promoter fragments and found that *C* allele of *-607A/C* or *G* allele of *-137G/C* showed higher activity of *IL-18*. The participants with *CC* homozygote of *-607A/C* or *GG* homozygote of *-137G/C* polymorphism exhibited somewhat higher levels of *IL-18* mRNA compared with other genotypes [9].

It is demonstrated that individuals exposure to environmental risk factors such as areca, alcohol, and tobacco consumption increase their susceptibility to oral cancer [10-13]. Also, genetic polymorphisms are reported to be the main risk factors of oral cancer susceptibility [14-16]. Our study suggested that *IL-18* polymorphisms *-607A/C* and *-137G/C* could regulate the protein levels of *IL-18* and considerably affect the individual sensitivity to oral cancer [9]. However, to the best of our knowledge, only Vairaktaris et al. [17] and Asefi et al. [18] have estimated the impact of *interleukin-18* polymorphisms *-607A/C* and *-137G/C* on oral cancer occurrence and clinical parameters. Among 238 Greek and German patients, Vairaktaris et al. recruited 149 with oral cancer and 89 who were healthy to examine the effect of *-607A/C* gene polymorphism of *IL-18* on oral cancer risk. They found that *IL-18 -607A/C* polymorphism is not associated with the susceptibility to oral cancer [17]. In Iran, Asefi et al. recruited 111 patients with head and neck squamous cell carcinoma and 212 who were healthy to investigate the association of *IL-18* gene polymorphisms *-607A/C* and *-137G/C* on the occurrence and clinical parameters of head and neck squamous cell carcinoma. Again, no meaningful association was found [18]. Unfortunately, their sample size limited the prediction of *IL-18 -607A/C* and *-137G/C* gene polymorphisms on the risk of oral cancer [17,18]. On the other hand, in a recently meta-analysis by Liang TJ et al. they found the *-137G > C* polymorphism significantly increased cancer risk in Asian population but not in Caucasian population after the stratification analyses of ethnicities [19]. We considered that the impact of genetic polymorphisms *IL-18 -607A/C* and *-137G/C* differences related to ethnicity, and their interaction with oral cancer related risk factor, including areca, tobacco, and alcohol consumption could increase oral cancer risk among Taiwanese. Therefore, for this study, we recruited 1,126 participants, including 567 patients with oral cancer and 559 healthy people to determine whether genetic variations at positions *-607A/C* and *-137G/C* of *IL-18* and their interaction with oral cancer-related risk factor are associated with the susceptibility to and clinicopathological development of oral cancer among Taiwanese people. 

## Materials and Methods

### Subjects and specimen collection

A total of 567 patients who were diagnosed with oral cancer, according to the characteristic criteria of national guidelines for oral cancer between April, 2007 and April, 2013 were recruited as a case group at Chung Shan Medical University Hospital in Taichung and Changhua Christian Hospital and Show Chwan Memorial Hospital in Changhua, Taiwan. Meanwhile, 559 resident area-, race-, and ethnic group-matched healthy individuals were randomly selected from the same geographic area to act as the controls. In addition, subjects with oral precancerous disease such as oral submucous fibrosis, leukoplakia, erythroplakia, verrucous hyperplasia, etc. were excluded from control group. For both cases and controls, we used a questionnaire to obtain exposure information about betel-nut chewing, tobacco use, and alcohol consumption. Medical information of the cases, including TNM clinical staging, the primary tumor size, lymph node involvement, and histologic grade, was obtained from their medical records. Oral-cancer patients were clinically staged at the time of their diagnosis according to the TNM staging system of the American Joint Committee on Cancer (AJCC) Staging Manual (7th ed.) [20]. Tumor differentiation was examined by a pathologist according to the AJCC classification. The whole blood specimens, collected from healthy controls and oral cancer patients, were placed in tubes containing EDTA and were immediately centrifuged and stored at -80 °C. The study was performed with the approval of the Chung Shan Medical University Hospital Institutional Review Board and informed written consent was obtained from each individual.

### Genomic DNA extraction

 Genomic DNA was extracted from whole blood samples collected from study subjects by QIAamp DNA blood mini kits (Qiagen, Valencia, USA) according to the manufacture's instructions. DNA was dissolved in TE buffer [10 mM Tris (pH 7.8), 1 mM EDTA] and then quantitated by a measurement of OD_260_. Final preparation was stored at −20 °C and used as templates in polymerase chain reaction (PCR) [21].

### Real-time PCR

Allelic discrimination of *-607A/C* (rs1946518) and *-137G/C* (rs187238) polymorphisms of the *IL-18* gene was assessed with the ABI StepOne™ Real-Time PCR System (Applied Biosystems, Foster City , CA , USA) and analyzed using SDS vers. 3.0 software (Applied Biosystems), with the TaqMan assay [22]. The primer sequences and probes for analysis of the *IL-18* gene polymorphisms are described in Table 1. The final volume for each reaction was 5 μL, containing 2.5 μL TaqMan Genotyping Master Mix, 0.125 μL TaqMan probe mix, and 10 ng genomic DNA. The real-time PCR included an initial denaturation step at 95 °C for 10 min, followed by 40 cycles of 95 °C for 15 s and 60 °C for 1 min. For each assay, appropriate controls (nontemplate and known genotype) were included in each typing run to monitor reagent contamination and as a quality control. To validate results from real-time PCR, around 5% of assays were repeated and several cases of each genotype were confirmed by the DNA sequence analysis.

**Table 1:** TaqMan primer sets for *IL-18* genotyped SNPs.

SNP	Probe
IL-18 -607A/C	VIC-5’- ATCATTAGAATTTTATTTAATAA
(rs1946518)	FAM-5’- TCATTAGAATTTTATGTAATAA
IL-18 -137G/C	VIC-5’- TCACTATTTTCATGAAATCTTTTCT
(rs187238)	FAM-5’-CACTATTTTCATGAAATGTTTTCT

### Statistical analysis

 Hardy–Weinberg equilibrium was assessed using a goodness-of-fit χ^2^ test for biallelic markers and estimated on Excel software. The average age are presented as the mean ± SE. The adjusted odds ratios (AORs) with their 95% confidence intervals (CIs) of the association between genotype frequencies and oral cancer risk as well as clinical characteristics were estimated by multiple logistic regression models after controlling for other covariates. A P value <0.05 was considered significant. The data were analyzed on SAS statistical software (Version 9.1, 2005; SAS Institute Inc., Cary, NC).

## Results

In our recruited control group, the frequencies of genetic polymorphisms such as *IL-18 -607 A/C* (p>0.05, χ^2^ value: 0.08) and *IL-18 -137 G/C* (p>0.05, χ^2^ value: 0.80) were in the Hardy-Weinberg equilibrium. 

The study estimated differences of demographical characteristics, such as gender, age, alcohol, tobacco, and areca consumption and genetic polymorphisms between oral cancer patients and controls. A significantly different distribution of *IL-18 -137 G/C* gene polymorphism based on gender, age, alcohol, tobacco, and areca consumption between oral cancer patients and controls was found (Table 2). To diminish the possible interference of environmental factors, adjusted ORs (AORs) with 95% CIs were estimated by multiple logistic regression models after controlling for other covariates in each comparison.

**Table 2:** The distributions of demographical characteristics and gene polymorphisms in 559 healthy controls and 567 patients with oral cancer.

Variable	Controls (n=559) (%)	Patients (n=567) (%)	p value
**Age (yrs**) Mean ± S.E.	51.86 ± 0.62	54.25 ± 0.47	**p=0.002**
**Gender**			
Male	456 (81.6%)	545 (96.1%)	
Female	103 (18.4%)	22 (3.9%)	**p<0.0001**
**Alcohol consumption**			
No	345 (61.7%)	231 (40.7%)	
Yes	214 (38.3%)	336 (59.3%)	**p<0.0001**
**Tobacco consumption**			
No	339 (60.6%)	85 (15.0%)	
Yes	220 (39.4%)	482 (85.0%)	**p<0.0001**
**Areca consumption**			
No	466 (83.4%)	134 (23.6%)	
Yes	93 (16.6%)	433 (76.4%)	**p<0.0001**
***IL-18 -607***			
***CC***	135 (24.1%)	140 (24.7%)	
***AC***	276 (49.4%)	262 (46.2%)	
***AA***	148 (26.5%)	165 (29.1%)	p=0.51
***IL-18 -137***			
***GG***	476 (85.2%)	437 (77.1%)	
***GC***	78 (13.9%)	122 (21.5%)	
***CC***	5 (0.9%)	8 (1.4%)	**p=0.002**
**Stage**			
I+II		248 (43.7%)	
III+IV		319 (56.2%)	
**Tumor T status**			
≤T2		348 (61.4%)	
T2		219 (38.6%)	
**Lymph node status**			
N0		357 (63.0%)	
N1+N2		210 (37.0%)	
**Metastasis**			
M0		559 (98.6%)	
M1		8 (1.4%)	
**Cell differentiated grade**			
≤Grade I		75 (13.2%)	
Grade I		492 (86.8%)	

An independent t-test or χ^2^ exact tests was used between healthy controls and patients with oral cancer.

People with *G/C* alleles of *IL-18 -137G/C* polymorphism had a 1.64-fold (95% CI=1.08-2.48; p=0.02) increased risk of developing oral cancer compared with those with *G/G* homozygotes. This determination was made after adjusting for gender, age, alcohol, tobacco, and areca consumption. However, there was not a significant association between *IL-18 -607A/C* genetic polymorphism and oral cancer. In addition, we found no gene-to-gene interaction effect on the increased susceptibility to oral cancer (Table 3). 

**Table 3:** Adjusted odds ratio (AOR) and 95% confidence intervals (CIs) of oral cancer associated with genotypic frequencies of *IL-18 -607A/C* and *IL-18 -137G/C*.

Variable	Controls (n=559) (%)	Patients (n=567) (%)	AOR (95% CI)	p value
***IL-18 -607***				
***CC***	135 (24.1%)	140 (24.7%)	1.00	
***AC***	276 (49.4%)	262 (46.2%)	0.91 (0.62-1.34)	p=0.65
***AA***	148 (26.5%)	165 (29.1%)	1.04 (0.67-1.60)	p=0.84
***IL-18 -137***				
***GG***	476 (85.2%)	437 (77.1%)	1.00	
***GC***	78 (13.9%)	122 (21.5%)	1.64 (1.08-2.48)	**p=0.02**
***CC***	5 (0.9%)	8 (1.4%)	0.89 (0.21-3.68)	p=0.88
***IL-18* genes combination**				
Group 1	131 (23.4%)	139 (24.5%)	1.00	
Group 2	349 (62.4%)	299 (52.7%)	0.81 (0.55-1.19)	p=0.29
Group 3	79 (14.2%)	129 (22.8%)	1.43 (0.87-2.33)	p=0.15

The odds ratios (ORs) with their 95% confidence intervals (CIs) were estimated by logistic regression models.

The adjusted odds ratios (AORs) with their 95% confidence intervals (CIs) were estimated by multiple logistic regression models, after controlling for gender, age, alcohol, tobacco, and areca consumption.

Group 1: individuals with *CC* of *IL-18 -607* and *GG* of *IL-18 -137*; Group 2: individuals with at least one of the following, including *A/C* or *A/A* of *IL-18 -607*, or *G/C* or *C/C* of *IL-18 -137*; Group 3: individuals with *A/C* or *A/A* of *IL-18 -607*, and *G/C* or *C/C* of *IL-18 -137*.

The study also determined whether there was an interaction effect of gene-to-related-environmental-risk-factors on oral cancer susceptibility. The adjusted odd ratios and 95% confidence intervals of genotypic frequencies and oral cancer susceptibility were estimated among persons with exposure and non-exposure to oral cancer-related environmental risk factors, respectively. There was no significant association between genetic polymorphisms of *IL-18 -607A/C* and *-137G/C* and oral cancer susceptibility among participants who had no exposure to related environmental risk factors (Table 4). However, among participants who were exposed to related environmental risk factors, including areca, alcohol, and tobacco consumption, the adjusted odd ratios and 95% confidence intervals were increased to a 2.02-fold (95% CI=1.01-4.04; p=0.04), 4.04-fold (95% CI=1.65-9.87; p=0.002), and 1.66-fold (95% CI=1.00-2.84; p=0.05) risk of developing oral cancer. For *-607A/C* polymorphism of *IL-18*, among alcohol consumers, those with *A/A* homozygotes of *IL-18 -607 A/C* polymorphism had a 2.38-fold (95% CI=1.17-4.86; p=0.01) increased risk of developing oral cancer compared with those with *C/C* homozygotes (Table 5). For gene-to-gene interaction effect, among alcohol consumers, those with group 3 polymorphism had a 5.81 (95% CI=2.22-15.24; p=0.0003) increased risk of developing oral cancer compared with those with group 1 (Table 5). This was determined after adjusting for confounders.

**Table 4:** Adjusted odds ratio (AOR) and 95% confidence intervals (CIs) of oral cancer associated with genotypic frequencies of *IL-18 -607A/C* and *IL-18 -137G/C* among individuals non-exposure to related environmental risk factors.

Variable	Controls	Patients	AOR (95% CI)	p value
**Among non-areca consumption** (**n=600**)				
***IL-18 -607***	Control (n=466) (%)	Case (n=134) (%)	AOR (95% CI)	p value
***CC***	114 (24.5%)	27 (20.2%)	1.00	
***AC***	226 (48.5%)	72 (53.7%)	1.12 (0.66-1.90)	p=0.65
***AA***	126 (27.0%)	35 (26.1%)	1.03 (0.57-1.86)	p=0.91
***IL-18 -137***				
***GG***	397 (85.2%)	107 (79.9%)	1.00	
***GC***	66 (14.2%)	26 (19.4%)	1.41 (0.82-2.41)	p=0.21
***CC***	3 (0.6%)	1 (0.7%)	1.32 (0.13-13.08)	p=0.80
***IL-18* genes combination**				
Group 1	111 (23.8%)	27 (20.2%)	1.00	
Group 2	289 (62.0%)	80 (59.6%)	0.97 (0.58-1.62)	p=0.91
Group 3	66 (14.2%)	27 (20.2%)	1.43 (0.74-2.74)	p=0.27
**Among non-alcohol consumption** (**n=576**)				
***IL-18 -607***	Control (n=345) (%)	Case (n=231) (%)	AOR (95% CI)	p value
***CC***	75 (21.7%)	59 (25.5%)	1.00	
***AC***	172 (49.9%)	103 (44.6%)	0.59 (0.34-1.01)	p=0.06
***AA***	98 (28.4%0	69 (29.9%)	0.63 (0.34-1.14)	p=0.12
***IL-18 -137***				
***GG***	277 (80.3%)	170 (73.6%)	1.00	
***GC***	64 (18.5%)	59 (25.5%)	1.19 (0.71-2.01)	p=0.50
***CC***	4 (1.2%)	2 (0.9%)	0.26 (0.03-1.85)	p=0.17
***IL-18* genes combination**				
Group 1	71 (20.6%)	59 (25.5%)	1.00	
Group 2	210 (60.9%)	111 (48.1%)	0.51 (0.30-0.87)	**p=0.01**
Group 3	64 (18.5%)	61 (26.4%)	0.72 (0.38-1.37)	p=0.32
**Among non-tobacco consumption** (**n=424**)				
***IL-18 -607***	Control (n=339) (%)	Case (n=85) (%)	AOR (95% CI)	P value
***CC***	76 (22.4%)	17 (20.0%)	1.00	
***AC***	169 (49.9%)	43 (50.6%)	0.89 (0.45-1.76)	p=0.74
***AA***	94 (27.7%)	25 (29.4%)	0.93 (0.43-1.99)	p=0.85
***IL-18 -137***				
***GG***	291 (85.8%)	65 (76.5%)	1.00	
***GC***	45 (13.3%)	20 (23.5%)	1.73 (0.86-3.45)	p=0.12
***CC***	3 (0.9%)	0 (0%)	─	p=0.98
***IL-18* genes combination**				
Group 1	74 (21.8%)	171 (20.0%)	1.00	
Group 2	219 (64.6%)	48 (56.5%)	0.78 (0.40-1.52)	p=0.47
Group 3	46 (13.6%)	20 (23.5%)	1.36 (0.59-3.16)	p=0.46

The odds ratios (ORs) with their 95% confidence intervals (CIs) were estimated by logistic regression models.

The adjusted odds ratios (AORs) with their 95% confidence intervals (CIs) were estimated by multiple logistic regression models, after controlling for gender, age, alcohol, tobacco, and areca consumption.

Group 1: individuals with *CC* of *IL-18 -607* and *GG* of *IL-18 -137*; Group 2: individuals with at least one of the following, including *A/C* or *A/A* of *IL-18 -607*, or *G/C* or *C/C* of *IL-18 -137*; Group 3: individuals with *A/C* or *A/A* of *IL-18 -607*, and *G/C* or *C/C* of *IL-18 -137*.

**Table 5:** Adjusted odds ratio (AOR) and 95% confidence intervals (CIs) of oral cancer associated with genotypic frequencies of *IL-18 -607 A/C* and *IL-18 -137 G/C* among individuals exposure to related environmental risk factors.

Variable	Controls	Patients	AOR (95% CI)	p value
**Among areca consumption** (**n=526**)				
***IL-18 -607***	Control (n=93) (%)	Case (n=433) (%)	AOR (95% CI)	p value
***CC***	21 (22.5%)	113 (26.1%)	1.00	
***AC***	50 (53.8%)	190 (43.9%)	0.78 (0.43-1.42)	p=0.42
***AA***	22 (23.7%)	130 (30.3)	1.11 (0.56-2.21)	p=0.75
***IL-18 -137***				
***GG***	79 (84.9%)	330 (76.2%)	1.00	
***GC***	12 (12.9%)	96 (22.2%)	2.02 (1.01-4.04)	**p=0.04**
***CC***	2 (2.2%)	7 (1.6%)	0.81 (0.15-4.26)	p=0.79
***IL-18* genes combination**				
Group 1	20 (21.5%)	112 (25.9%)	1.00	
Group 2	60 (64.5%)	219 (50.6%)	0.70 (0.39-1.27)	p=0.24
Group 3	13 (14.0%)	102 (23.5%)	1.54 (0.69-3.41)	p=0.28
**Among alcohol consumption** (**n=550**)				
***IL-18 -607***	Control (n=214) (%)	Case (n=336) (%)	AOR (95% CI)	p value
***CC***	60 (28.0%)	81 (24.1%)	1.00	
***AC***	104 (48.6%)	159 (47.3%)	1.68 (0.93-3.03)	p=0.08
***AA***	50 (23.4%)	96 (28.6%)	2.38 (1.17-4.86)	**p=0.01**
***IL-18 -137***				
***GG***	199 (93.0%)	267 (79.5%)	1.00	
***GC***	14 (6.5%)	63 (18.7%)	4.04 (1.65-9.87)	**p=0.002**
***CC***	1 (0.5%)	6 (1.8%)	8.82 (0.48-161.7)	p=0.14
***IL-18* genes combination**				
Group 1	60 (28.0%)	80 (23.8%)	1.00	
Group 2	139 (65.0%)	188 (56.0%)	1.54 (0.87-2.72)	p=0.13
Group 3	15 (7.0%)	68 (20.2%)	5.81 (2.22-15.24)	**p=0.0003**
**Among tobacco consumption** (**n=702**)				
***IL-18 -607***	Control (n=220) (%)	Case (n=482) (%)	AOR (95% CI)	p value
***CC***	59 (26.8%)	123 (25.5%)	1.00	
***AC***	107 (48.6%)	219 (45.4%)	0.93 (0.57-1.51)	p=0.77
***AA***	54 (24.6%)	140 (29.1%)	1.20 (0.69-2.09)	p=0.51
***IL-18 -137***				
**GG**	185 (84.1%)	372 (77.2%)	1.00	
**GC**	33 (15.0%)	102 (21.2%)	1.66 (1.00-2.84)	**p=0.05**
***CC***	2 (0.9%)	8 (1.6%)	1.36 (0.23-7.82)	p=0.72
***IL-18* genes combination**				
Group 1	57 (25.9%)	122 (25.3%)	1.00	
Group 2	130 (59.1%)	251 (52.1%)	0.84 (0.52-1.36)	p=0.49
Group 3	33 (15.0%)	109 (22.6%)	1.55 (0.83-2.90)	p=0.16

The odds ratios (ORs) with their 95% confidence intervals (CIs) were estimated by logistic regression models.

The adjusted odds ratios (AORs) with their 95% confidence intervals (CIs) were estimated by multiple logistic regression models, after controlling for gender, age, alcohol, tobacco, and areca consumption.

Group 1: individuals with *CC* of *IL-18 -607* and *GG* of *IL-18 -137*; Group 2: individuals with at least one of the following, including *A/C* or *A/A* of *IL-18 -607*, or *G/C* or *C/C* of *IL-18 -137*; Group 3: individuals with *A/C* or *A/A* of *IL-18 -607*, and *G/C* or *C/C* of *IL-18 -137*.

Both genetic polymorphisms were analyzed with regard to the clinical status of each of our recruited 567 oral cancer patients, including the tumor stage, tumor size, lymph node metastasis, distant metastasis, and cancer cell differentiation. Patients with *G/C* alleles *IL-18 -137G/C* polymorphism showed a decreased risk of developing Stages III-IV (AOR=0.59; 95% CI=0.39-0.89; p=0.01), a tumor size > T2 (AOR=0.56; 95% CI=0.35-0.87; p=0.01), and lymph node metastasis (AOR=0.51; 95% CI=0.32-0.80; p=0.003). There was not a significant association between clinical status and *IL-18 -607 A/C* gene polymorphism in these patients (Table 6). 

**Table 6:** Adjusted odds ratio (AOR) and 95% confidence intervals (CI) of clinical statuses associated with genotypic frequencies of *IL-18 -607A/C* and *IL-18-137* *G/C* in oral cancer patients (n=567).

**Clinical Stage**				
***IL-18 -607***	Stage < III (n=248) (%)	Stage ≥ III (n=319) (%)	AOR (95% CI)	p value
***CC***	63 (25.4%)	77 (24.1%)	1.00	
***AC***	120 (48.4%)	142 (44.5%)	0.95 (0.62-1.44)	p=0.81
***AA***	65 (26.2%)	100 (31.4%)	1.25 (0.78-1.98)	p=0.34
***IL-18 -137***	Stage < III (n=248) (%)	Stage ≥ III (n=319) (%)	AOR (95% CI)	p value
***GG***	180 (72.6%)	257 (80.6%)	1.00	
***GC***	67 (27.0%)	55 (17.2%)	0.59 (0.39-0.89)	**p=0.01**
***CC***	1 (0.4%)	7 (2.2%)	4.58 (0.55-37.66)	p=0.15
**Tumor size**				
***IL-18 -607***	≤ T2 (n=348) (%)	> T2 (n=219) (%)	AOR (95% CI)	p value
***CC***	90 (25.9%)	50 (22.8%)	1.00	
***AC***	166 (47.7%)	96 (43.9%)	1.02 (0.66-1.57)	p=0.92
***AA***	92 (26.4%)	73 (33.3%)	1.40 (0.88-2.24)	p=0.14
***IL-18 -137***	≤ T2 (n=348) (%)	> T2 (n=219) (%)	AOR (95% CI)	P value
***GG***	257 (73.8%)	180 (82.2%)	1.00	
***GC***	88 (25.3%)	34 (15.5%)	0.56 (0.35-0.87)	**p=0.01**
***CC***	3 (0.9%)	5 (2.3%)	2.30 (0.54-9.81)	p=0.25
**Lymph node metastasis**				
***IL-18 -607***	No (n=357) (%)	Yes (n=210) (%)	AOR (95% CI)	p value
***CC***	85 (23.8%)	55 (26.2%)	1.00	
***AC***	172 (48.2%)	90 (42.9%)	0.80 (0.52-1.23)	p=0.31
***AA***	100 (28.0%)	65 (30.9%)	1.00 (0.63-1.60)	p=0.97
***IL-18 -137***	No (n=357) (%)	Yes (n=210) (%)	AOR (95% CI)	P value
***GG***	261 (73.1%)	176 (83.8%)	1.00	
***GC***	91 (25.5%)	31 (14.8%)	0.51 (0.32-0.80)	**p=0.003**
***CC***	5 (1.4%)	3 (1.4%)	0.85 (0.20-3.65)	p=0.83
**Distant metastasis**				
***IL-18 -607***	No (n=559) (%)	Yes (n=8) (%)	AOR (95% CI)	p value
***CC***	137 (24.5%)	3 (37.5%)	1.00	
***AC***	259 (46.3%)	3 (37.5%)	0.43 (0.08-2.27)	p=0.32
***AA***	163 (29.2%)	2 (25%)	0.49 (0.08-3.08)	p=0.45
***IL-18 -137***	No (n=559) (%)	Yes (n=8) (%)	AOR (95% CI)	P value
***GG***	429 (76.8%)	8 (100%)	1.00	
***GC***	122 (21.8%)	0 (0%)	─	p=0.94
***CC***	8 (1.4%)	0 (0%)	─	p=0.98
**Cell differentiated grade**				
***IL-18 -607***	≦Grade I (n=75) (%)	Grade I (n=492) (%)	AOR (95% CI)	p value
***CC***	19 (25.3%)	121 (24.6%)	1.00	
***AC***	39 (52.0%)	223 (45.3%)	0.89 (0.49-1.63)	p=0.72
***AA***	17 (22.7%)	148 (30.1%)	1.37 (0.68-2.77)	p=0.37
***IL-18 -137***	≦Grade I (n=75) (%)	Grade I (n=492) (%)	AOR (95% CI)	p value
***GG***	61 (81.3%)	376 (76.4%)	1.00	
***GC***	14 (18.7%)	108 (22.0%)	1.24 (0.66-2.32)	p=0.48
***CC***	0	8 (1.6%)	─	p=0.98

The odds ratios (ORs) with their 95% confidence intervals (CIs) were estimated by logistic regression models.

The adjusted odds ratios (AORs) with their 95% confidence intervals (CIs) were estimated by multiple logistic regression models, after controlling for gender, age, alcohol, tobacco, and areca consumption.

> T2: multiple tumor more than 2 cm. Cell differentiate grade: grade I: well differentiated; grade II: moderately differentiated; grade III: poorly differentiated.

## Discussion

 Our study offered information that *IL-18* gene promoter polymorphism *-137G/C* was significantly associated with oral cancer susceptibility and clinicopathological development. 

*IL-18* has been shown to act as a regulator of oral cancer development [5,8]. Hayes et al. suggested that oral talactoferrin, a recombinant human lactoferrin, produced a dose-dependent inhibition of oral tumors through an increased expression of *IL-18* [8]. Jablonska et al. observed a considerably lower concentration of *IL-18* released by polymorphonuclear leukocytes (PMN) derived from oral cavity cancer patients when compared with those of healthy people. However, the production of *IL-18* by PMN was enhanced among oral carcinoma patients after cancer treatment. In our study, participants with *G/C* alleles of *IL-18 -137 G/C* polymorphism had a 1.64-fold (95% CI=1.08-2.48; p=0.02) increased risk of developing oral cancer compared with participants with *G/G* homozygotes, a determination made after adjusting for gender; age; and alcohol, tobacco, and areca consumption, our result was inconsistent with those of Vairaktaris et al [17] and Asefi et al. [18]., However, for *-607A/C* polymorphism of *IL-18*, our results were similar to those of Vairaktaris et al and Asefi et al., which indicated there was not a significant relationship between *IL-18 -607A/C* polymorphism and oral cancer risk [17,18]. We suggest that *C* allele of *IL-18 -137G/C* polymorphisms lead to a lower level of *IL-18* protein synthesis [9]. Such an occurrence impedes the modulation of cell cycle arrest and the triggering of cell apoptosis, which protects the host from oral cancer development [4,6-8]. Moreover, the inconsistent results between ours and those of Vairaktaris et al and Asefi et al., indicating the impaction of genetic polymorphism *-137G/C* on oral cancer susceptibility may be difference related to ethnicity [19]. 

The exposure of patients to oral cancer-related environmental risk factors such as areca, alcohol, and tobacco consumption demonstrate an increased risk to cause mucosal fibroblast proliferation and oral epithelial hyperplasia and dysplasia, in which cancer-related tissue chronic inflammation is suggested involved [10-13,23-26]. In our study finds that the interaction of gene to oral cancer-related environmental risk factors has a synergetic effect that can further enhance oral cancer development. Among participants exposed to oral cancer-related environmental risk factors, including areca, alcohol, and tobacco consumption. the adjusted odd ratios and 95% confidence intervals increased to a 2.02-fold (95% CI=1.01-4.04; p=0.04), 4.04-fold (95% CI=1.65-9.87; p=0.002), and 1.66-fold (95% CI=1.00-2.84; p=0.05) risk of developing oral cancer for participants with *G/C* alleles of *IL-18 -137 G/C* polymorphism compared to participants with *G/G* homozygotes. Also, among alcohol consumers, participants with *A/A* homozygotes of *IL-18 -607 A/C* polymorphism had a 2.38-fold (95% CI=1.17-4.86; p=0.01) increased risk of developing oral cancer compared with participants with *C/C* homozygotes, determined after adjusting for confounders. It was known that treatment of ovalbumin-sensitized mice with areca nut extract significantly augmented inflammatory response and promoted the development of CD 11b^+^ Gr-1^+^ cells with the characteristics of myeloid-derived suppressor cells, which could skew the host immunity toward tumor-promotion and deteriorate anti-tumor immunity by down-regulating T-cell reactivity to cancer cells [25,26]. Tobacco is a heterogeneous which contains different substances classified as carcinogenic to human [27,28]. Tobacco consumption has been linked to the development of cancer-related inflammation in cancer patients [29] and to induction of oral epithelial hyperplasia and dysplasia among patients with oral cancer [23,24]. Recently, cancer-related inflammation is mentioned as one of the cancer hallmarks, because it involved in the initiation of genetic instability by inflammatory mediators [25,26,30]. We suggested that both areca nut and tobacco consumption could amplify inflammatory response and long term consumption of either areca nut or tobacco smoke could induce chronic inflammation of oral tissue, which subsequently lead to accumulation of random genetic alteration and initiate the development of oral cancer [25,26,30]. *IL-18* exerts its anti-tumor activity by inducing cell cycle arrest for DNA repair, promoting cytotoxic cells activity, and triggering mutated cells apoptosis [4,6-8]. However, among individuals with *C* allele of *IL-18 -137G/C* polymorphisms result in a lower level of *IL-18* protein production [9], consequently, damaged DNA does not repaired due to the fail of inducing cell cycle arrest and the defeat of triggering mutated cell apoptosis by *IL-18*, genetically damaged cells proliferate, giving rise eventually increase the risk to malignant neoplasm among subjects with *C* allele of *IL-18 -137G/C* polymorphisms [4,6-8]. Also, alcohol consumption is shown to modulate adaptive immune responses and inflammatory process [31,32]. Joosten et al. estimated gene expression profiles of leucocytes and circulating proteins related to immune response after moderate alcohol consumption among twenty-four healthy men. They found plasma levels of pro-inflammatory IL-1 receptor antagonist and *IL-18* significantly decreased after alcohol consumption [32]. We suggested that alcohol consumption induce decreased expression of *IL-18* and result in abating the function of *IL-18* on the modulation of cell-cycle arrest and induction of apoptosis, particularly to the subjects with *A* allele of *IL-18 -607* or *C* allele of *IL-18 -137* polymorphisms because both genetic polymorphisms are suggested with lower activity of *IL-18*, consequently enhance the risk to have oral cancer among alcohol consumers with *A* allele of *IL-18 -607* or *C* allele of *IL-18 -137G/C* gene polymorphisms. 

However, we also found that *G/C* genotype *IL-18 -137* polymorphism represented a protective factor for oral cancer progression. Patients with *G/C* alleles of *IL-18 -137* correlated with a lower clinical stage, tumor size, and non-lymph node metastasis compared with patients with *G/G* alleles. Our results were similar to those of Jaiswal et al. [33] and Saenz-Lopez et al. [34]. Jaiswal et al. [33] recruited 200 patients with bladder cancer and 200 healthy controls to examine the impact of *IL-18* gene polymorphism on bladder cancer susceptibility, they found a significant relationship of *IL-18 -137 G/C* heterozygous genotype (*G/C*) with 1.96 folds risk to bladder cancer, however, this heterozygous genotype showed a significantly reduced risk for tumor progression among patients with bladder cancer. Also, Saenz-Lopez et al. [34] found that *IL-18 -137 GG* genotype was significantly associated with a higher tumor size, grade, and stage among patients with renal cell carcinoma. It is found that significantly higher levels of *IL-18* in serum and culture supernatants of PMN from patients with oral cancer in Stages III and IV as compared with patients in Stages I and II [5]. Our study suggests that the over-expression of *IL-18* plays a vital role in protecting people from oral cancer. However, the higher concentrations of *IL-18* in serum and culture supernatants of PMN from patients with oral cancer in Stages III and IV as compared with patients in Stages I and II could be the host’s response against the growth and progression of oral cancer. Furthermore, it is probable that *IL-18* acts as both a suppressor and promoter in the regulation of oral cancer development [5,35-37]. One of the possible explanations for the controversial effect of *IL-18 -137 G/C* polymorphism in oral cancer susceptibility and clinical progression is that *IL-18* has dual effects on cancer development and progression [5,37-39]. Studies have suggested that *IL-18* plays a major role in angiogenesis. Specifically, it has been reported that malignant cancer cells increase their adherence to microvascular wall and even promote production of angiogenic and tumor growth-stimulating factor through the *IL-18*-dependendent pathway [38,39]. Over-expression of *IL-18* was found among cancer patients with malignant prognosis, including oral cancer, lung cancer, gastric cancer, pancreatic cancer, and hepatocellular carcinoma [5,37,40-42]. We suggest that *IL-18* can act against the occurrence of oral cancer and induce angiogenesis and metastasis, which, in part, play a role in the advanced progression of oral cancer. Our study also demonstrates that patients with *G/C* alleles of *IL-18 -137 G/C* polymorphism can express lower levels of *IL-18* compared with patients with *G/G* homozygotes, which benefits the inhibition of angiogenesis and tumor growth and consequently protects patients from the progression of oral cancer [5,9,37]. 

In conclusion, our results suggest that *IL-18 -137 G/C* gene polymorphism may be a factor that increases the susceptibility to oral cancer and can be a protective factor against oral cancer progression. The interactions of gene to oral cancer-related environmental risk factors have a synergetic effect that can further enhance oral cancer development.
